# Red Ginseng Ethanolic Extract Alleviates DSS-Induced Colitis in Mice by Suppressing Inflammatory Mediator Production

**DOI:** 10.3390/ijms27125325

**Published:** 2026-06-12

**Authors:** Peng-Yu Zhang, Wen-Yu Yu, Ke-Xin Zhang, Xing-Hao Jin, Yi-Dong Song, Mei-Lan Lian, Yue-Jun Hao, Jun Jiang

**Affiliations:** 1 Key Laboratory of Natural Medicines of the Changbai Mountain, Ministry of Education, Yanbian University, Park Road 977, Yanji 133002, China; zhang13160522@163.com (P.-Y.Z.); 18242441913@163.com (W.-Y.Y.); aiskexin@163.com (K.-X.Z.); 0000008094@ybu.edu.cn (X.-H.J.); lianmeilan2001@163.com (M.-L.L.); 2 Yanbian Prefecture Agricultural Biological Resources and Rural Energy Management Station, Tianchi Road 3418, Yanji 133000, China

**Keywords:** red ginseng ethanol extract, ulcerative colitis, DSS-induced colitis, RAW 264.7 macrophages, inflammatory mediators, ginsenosides

## Abstract

Ulcerative colitis (UC) is a chronic inflammatory bowel disease characterized by recurrent intestinal inflammation and mucosal injury. This study evaluated the protective potential of red ginseng ethanolic extract (RGEE) using a dextran sulfate sodium (DSS)-induced colitis mouse model and an LPS-stimulated RAW 264.7 macrophage model. Preliminary LC-MS profiling was also performed to characterize the detectable chemical features of RGEE. In vivo, RGEE alleviated DSS-induced body weight loss, disease activity, colon shortening, spleen enlargement, and histopathological injury, with the histopathological score reduced by approximately 51.1%. RGEE also partially improved DSS-induced hematological alterations without causing obvious changes in major organ weights. In vitro, RGEE showed no obvious cytotoxicity up to 250 μg/mL and reduced LPS-induced NO, TNF-α, IL-6, and IL-1β production by approximately 60.0–67.1%. LC-MS analysis putatively annotated several saponin-related features, including notoginsenoside R1 and ginsenosides Rb1, Rb2, Rh1, Rh4, and Rh2. These findings suggest that RGEE has protective potential against DSS-induced colitis, which is associated with the suppression of inflammatory mediator production. Further studies are needed to clarify its active constituents and mechanisms of action.

## 1. Introduction

Ulcerative colitis (UC) is a chronic, relapsing inflammatory disease of the colon and rectum characterized by mucosal inflammation, abdominal pain, diarrhea, rectal bleeding, and recurrent disease activity [1]. In recent years, the global burden of UC has continued to increase, with an estimated prevalence of approximately 5 million cases worldwide in 2023 [2]. The rising incidence of UC, particularly in newly industrialized regions, highlights an urgent need for safer, accessible, and long-term applicable strategies for disease prevention and management [3,4,5]. Although current therapies, including aminosalicylates, corticosteroids, immunosuppressants, and biological agents, have improved clinical outcomes, their long-term use is still limited by variable efficacy, relapse, adverse effects, and economic burden [4,5,6,7]. Therefore, the discovery of natural products with anti-inflammatory activity and good safety profiles has become an important direction for developing complementary interventions against UC.

The pathogenesis of UC is complex and involves epithelial barrier disruption, dysregulated immune responses, oxidative stress, gut microbiota imbalance, and excessive production of inflammatory mediators [8,9]. Among these factors, macrophage-mediated inflammatory responses play a critical role in the amplification of intestinal inflammation. Activated macrophages can produce nitric oxide and pro-inflammatory cytokines such as tumor necrosis factor-α, interleukin-6, and interleukin-1β, which further aggravate mucosal injury and inflammatory progression [10,11]. Therefore, inhibition of inflammatory mediator production represents an important strategy for alleviating UC-related intestinal damage. The dextran sulfate sodium (DSS)-induced colitis mouse model is widely used for UC research because it reproduces several pathological and clinical features of human UC, including body weight loss, diarrhea, rectal bleeding, colon shortening, and mucosal inflammation [12,13]. Meanwhile, lipopolysaccharide (LPS)-stimulated RAW 264.7 macrophages provide a useful in vitro model for evaluating anti-inflammatory activity [14,15]. Although DSS-induced colitis and RAW 264.7 macrophages are useful models for the preliminary evaluation of RGEE, they cannot fully reproduce the chronic, multifactorial immunopathology of human ulcerative colitis, particularly adaptive immune dysregulation and microbiota–host interactions; therefore, further validation in more physiologically relevant systems is needed.

Red ginseng, a steamed and processed form of *Panax ginseng*, has long been used as a functional food and traditional medicinal material in East Asia [16,17]. Red ginseng contains multiple bioactive constituents, including ginsenosides, phenolic compounds, polysaccharides, and other secondary metabolites, which are associated with anti-inflammatory, antioxidant, and immunomodulatory activities [18,19,20]. Increasing evidence suggests that ginseng and its active components may exert protective effects against intestinal inflammation by regulating inflammatory cytokines, oxidative stress, epithelial barrier function, and immune homeostasis [21,22,23]. Previous studies have reported that ginsenosides (including Rh2, Rh1, Rg1, Rb1, Rc, and Rf) and red ginseng-derived preparations (including red ginseng powder, aqueous extracts, concentrated extracts, capsules or tablets, beverages, fermented red ginseng, and related dietary supplements or functional food products) can alleviate experimental colitis, reduce pro-inflammatory mediator production, and improve colitis-associated pathological symptoms [6,24].

However, most existing studies have focused on purified ginsenosides, fermented red ginseng, red ginseng-derived nanoparticles, or compound herbal formulations. In contrast, red ginseng ethanol extract (RGEE), as a multi-component crude extract, is more representative of functional food or dietary supplement-type preparations. The use of ethanol extraction is simple, scalable, and suitable for enriching multiple ginsenosides and phenolic constituents. Compared with single compounds, crude extracts may exert integrated anti-inflammatory effects through multi-component and multi-target actions [20,25]. Nevertheless, whether red ginseng ethanol extract can effectively alleviate UC symptoms and suppress macrophage-mediated inflammatory responses remains insufficiently clarified.

In this study, we investigated the protective effects of RGEE against UC using both in vivo and in vitro experimental models. DSS-induced colitis mice were used to evaluate the effects of RGEE pretreatment on water and food intake, body weight change, disease activity index (DAI), rectal bleeding, colon length, hematological parameters, and organ weights. In parallel, LPS-stimulated RAW 264.7 cells were used to assess the inhibitory effects of RGEE on nitric oxide (NO) production and pro-inflammatory cytokine secretion. This integrated evaluation provides experimental evidence for the anti-colitis activity, anti-inflammatory potential, and preliminary safety of RGEE, supporting its possible application as a functional food-derived candidate for the prevention or alleviation of UC.

## 2. Results

### 2.1. Preliminary Chemical Profiling of RGEE by LC-MS Analysis

LC-MS analysis was performed to obtain a preliminary chemical profile of RGEE. A representative total ion chromatogram is shown in Figure S1. As summarized in Table 1, 12 saponin-related LC-MS features were screened based on retention time, predicted molecular formula, adduct ions, measured *m*/*z* values, and database matching results, with a mass accuracy tolerance of 5 ppm. Among them, several features were putatively annotated as Panax-type saponins, including notoginsenoside R1 (C_47_H_80_O_18_), ginsenoside Rb1 (C_54_H_92_O_23_), ginsenoside Rb2 (C_53_H_90_O_22_), 20(R)-ginsenoside Rh1 (C_36_H_62_O_9_), ginsenoside Rh4 (C_36_H_60_O_8_), and 20(R)-ginsenoside Rh2 (C_36_H_62_O_8_). Because these assignments were not confirmed using authentic standards, they should be regarded as putative annotations rather than definitive identifications. These results indicate that RGEE contains multiple saponin-related LC-MS features, providing a preliminary chemical basis for further investigation of its anti-colitis effects.

### 2.2. Protective Effects of RGEE Against DSS-Induced Colitis in Mice

#### 2.2.1. Effects of RGEE on Water Intake, Food Intake, Body Weight Change, and Disease Activity Index

**Figure 1:**
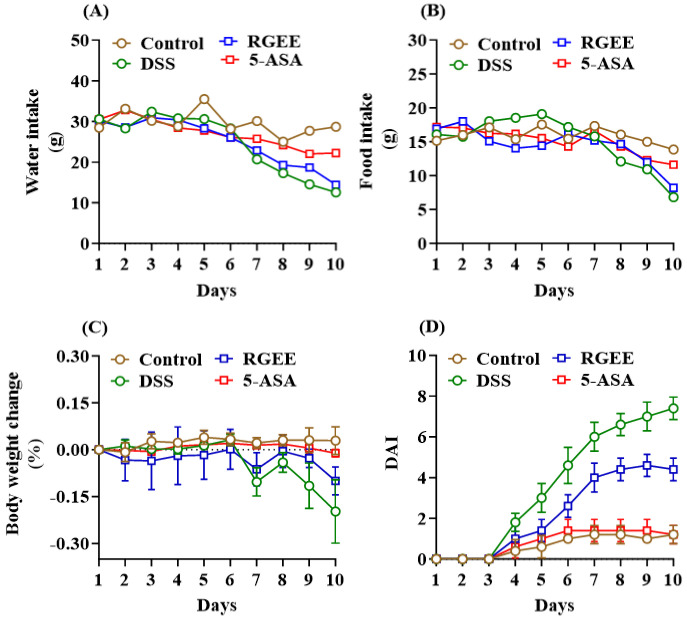
Effects of red ginseng ethanolic extract (RGEE) pretreatment on water intake, food intake, body weight change, and disease activity index in DSS-induced colitis mice. (**A**) Water intake. (**B**) Food intake. (**C**) Body weight change. (**D**) Disease activity index (DAI). DSS, DSS-induced colitis model group; 5-ASA, positive control group. Data are presented as the mean ± SD (n = 10).

As shown in Figure 1, DSS treatment affected the general physiological condition of mice. During the early experimental period, water intake, food intake, body weight change, and disease activity index (DAI) showed no obvious differences among the groups. However, after DSS induction, mice in the DSS model group exhibited decreased water and food intake, progressive body weight loss, and a rapid increase in DAI, indicating the successful establishment of DSS-induced colitis.

Compared with the DSS group, RGEE pretreatment alleviated the decline in water and food intake, suggesting a partial improvement in the general health status of DSS-treated mice. RGEE pretreatment also reduced the extent of body weight loss compared with the DSS group, although body weight did not fully return to the level of the Control group. Notably, the DAI score increased sharply in the DSS group from day 4 onward, whereas RGEE pretreatment clearly suppressed the increase in DAI during the later stage of the experiment. The 5-ASA group, used as the positive control, showed a more pronounced protective effect, with relatively stable food intake, body weight, and a markedly lower DAI score compared with the DSS group. These results suggest that RGEE pretreatment ameliorated DSS-induced colitis symptoms, as reflected by improved water and food intake, reduced body weight loss, and decreased disease activity.

#### 2.2.2. Effects of RGEE on Rectal Bleeding and Colon Length

**Figure 2:**
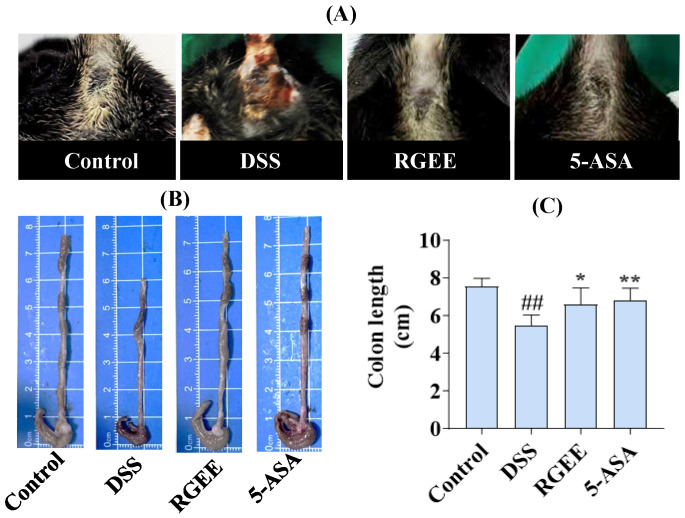
Effects of red ginseng ethanolic extract (RGEE) pretreatment on rectal bleeding and colon damage in DSS-induced colitis mice. (**A**) Representative images of rectal bleeding. (**B**) Representative images of the colon. (**C**) Colon length. DSS, DSS-induced colitis model group; 5-ASA, positive control group. Data are presented as the mean ± SD (n = 10). ## *p* < 0.01 vs. the Control group; * *p* < 0.05 and ** *p* < 0.01 vs. the DSS group.

As shown in Figure 2A, mice in the Control group showed no obvious rectal bleeding, whereas mice in the DSS group exhibited severe rectal bleeding in the perianal region, indicating the induction of colitis-related symptoms. Compared with the DSS group, RGEE pretreatment visibly alleviated rectal bleeding, and a similar protective effect was observed in the 5-ASA group. Colon shortening is an important macroscopic indicator of DSS-induced colitis. As shown in Figure 2B, C, DSS treatment significantly reduced colon length compared with the Control group (*p* < 0.001). In contrast, RGEE pretreatment significantly restored colon length compared with the DSS group (*p* < 0.05), suggesting that RGEE attenuated DSS-induced colon injury. The positive control, 5-ASA, also significantly increased colon length compared with the DSS group (*p* < 0.01). These results indicate that RGEE pretreatment has the potential to alleviate DSS-induced colitis.

#### 2.2.3. Effects of RGEE on Histopathological Injury in Colon Tissues

**Figure 3:**
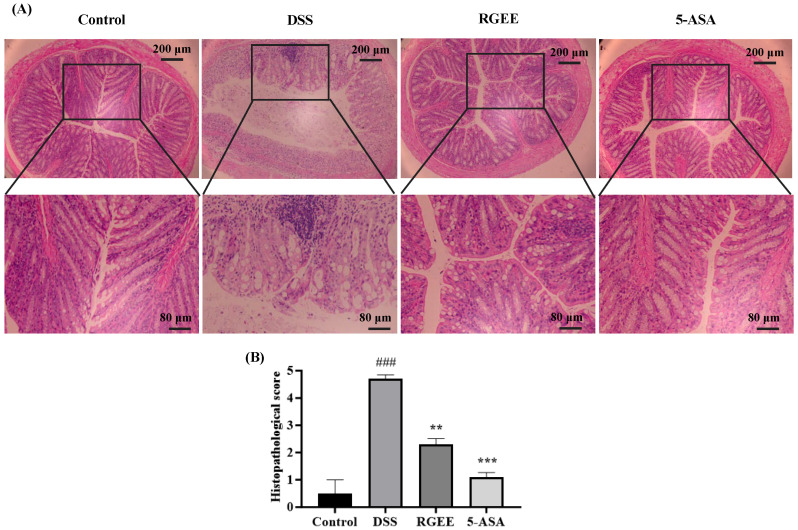
Effects of red ginseng ethanolic extract (RGEE) pretreatment on histopathological injury in colon tissues of DSS-induced colitis mice. (**A**) Representative hematoxylin and eosin -stained images of colon tissues. (**B**) Histopathological score. DSS, DSS-induced colitis model group; 5-ASA, positive control group. Data are presented as the mean ± SD (n = 10). ### indicates a significant difference compared with the control group at *p* < 0.001. ### *p* < 0.001 vs. the Control group; ** *p* < 0.01 and *** *p* < 0.001 vs. the DSS group.

Hematoxylin and eosin (H&E)-stained images of colon tissues showed that the Control group exhibited normal colonic architecture, with an intact mucosal structure, well-organized crypts, and no obvious inflammatory cell infiltration (Figure 3A). In contrast, the DSS group showed marked histopathological injury, including mucosal architecture disruption, crypt damage/loss, inflammatory cell infiltration, and epithelial destruction. These morphological changes were accompanied by a significantly higher histopathological score than that of the control group, supporting the successful establishment of DSS-induced colitis-related pathological injury. The RGEE-treated mice showed relatively preserved mucosal architecture, reduced crypt damage, and decreased inflammatory cell infiltration. A similar protective effect was observed in the 5-ASA group.

The histopathological score further confirmed the H&E staining observations. As shown in Figure 3B, DSS treatment significantly increased the histopathological score compared with the Control group (*p* < 0.001), whereas RGEE pretreatment significantly reduced the score compared with the DSS group (*p* < 0.001). The 5-ASA group also showed a significant reduction in histopathological score compared with the DSS group (*p* < 0.001).

#### 2.2.4. Effects of RGEE on Hematological Parameters

As shown in Table 2, DSS treatment altered several hematological parameters in mice. Compared with the Control group, the DSS group showed significantly increased levels of WBC, NEU, LYM, and MON, indicating an enhanced peripheral inflammatory response after DSS induction. In contrast, RGEE pretreatment significantly reduced the DSS-induced increases in WBC, NEU, LYM, and MON, with values closer to those observed in the Control group. A similar trend was observed in the 5-ASA group.

DSS treatment also significantly decreased RBC, HGB, and MCH levels compared with the Control group, suggesting that DSS-induced colitis may affect erythrocyte-related parameters. RGEE pretreatment significantly restored RBC and MCH levels compared with the DSS group, while HGB showed an increasing trend but did not reach statistical significance. In contrast, 5-ASA significantly restored RBC, HGB, and MCH levels compared with the DSS group. HCT, MCV, MCHC, and MPV showed no significant differences among the relevant comparisons.

For platelet-related parameters, PLT was significantly increased in the DSS group compared with the Control group, whereas RGEE and 5-ASA pretreatment significantly reduced PLT levels toward the normal range. These results suggest that RGEE pretreatment may alleviate DSS-induced hematological alterations, particularly by reducing inflammatory leukocyte responses and partially improving erythrocyte- and platelet-related parameters.

#### 2.2.5. Effects of RGEE on Organ Weights

**Figure 4:**
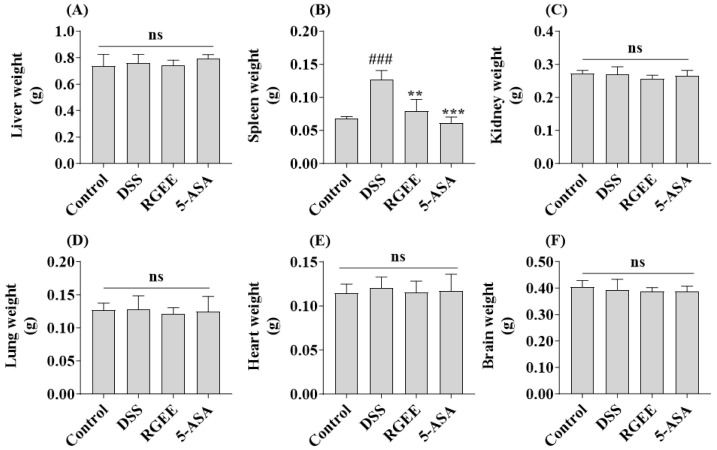
Effects of red ginseng ethanolic extract (RGEE) on organ weights in mice. (**A**) Liver weight. (**B**) Spleen weight. (**C**) Kidney weight. (**D**) Lung weight. (**E**) Heart weight. (**F**) Brain weight. DSS, DSS-induced colitis model group; 5-ASA, positive control group. Data are presented as the mean ± SD (n = 10). ns, not significant. ### *p* < 0.001 vs. the Control group; ** *p* < 0.01 and *** *p* < 0.001 vs. the DSS group.

As shown in Figure 4, DSS-induced colitis did not markedly affect the weights of most major organs, including the liver, kidneys, lungs, heart, and brain, as no significant differences were observed among the groups. In contrast, spleen weight was significantly increased in the DSS group compared with the Control group (*p* < 0.001), suggesting that DSS-induced colitis was associated with systemic immune activation.

Compared with the DSS group, RGEE pretreatment significantly reduced spleen weight (*p* < 0.01), indicating that RGEE alleviated DSS-induced spleen enlargement. Similarly, the positive control, 5-ASA, showed a significant reduction in spleen weight compared with the DSS group (*p* < 0.001). However, there were no significant differences in the weights of the liver, kidneys, lungs, heart and brain among the different groups. These results suggest that RGEE pretreatment may attenuate DSS-induced systemic inflammatory responses, as reflected by reduced spleen enlargement, without causing apparent changes in the weights of major organs.

### 2.3. Effects of RGEE on LPS-Induced Inflammatory Responses in RAW 264.7 Cells

**Figure 5:**
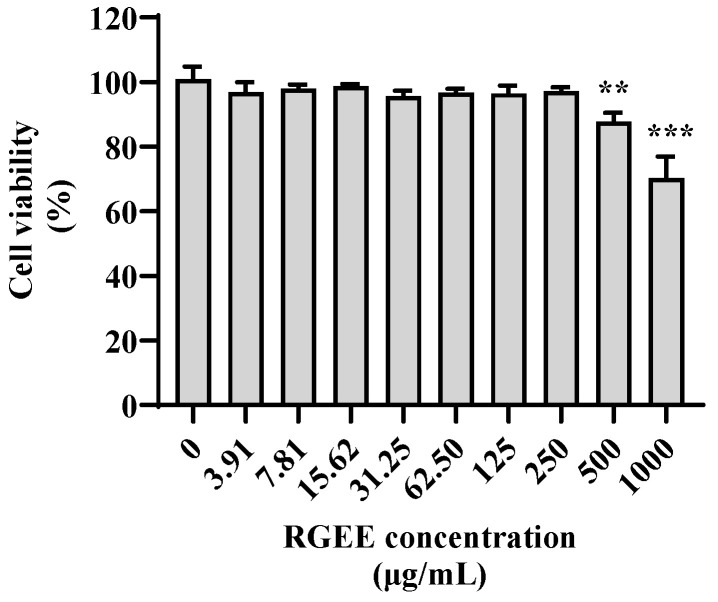
Effects of ginseng ethanolic extract (RGEE) on the viability of RAW 264.7 cells. Data are presented as the mean ± SD (n = 6). ** *p* < 0.01 and *** *p* < 0.001 vs. the control group (0 g/mL).

RGEE showed no obvious cytotoxicity toward RAW 264.7 cells at concentrations ranging from 3.91 to 250 μg/mL (Figure 5). However, cell viability was significantly reduced at 500 and 1000 μg/mL compared with the untreated control group, indicating that high concentrations of RGEE may exert cytotoxic effects. Therefore, non-cytotoxic concentrations of RGEE, including 62.5, 125, and 250 μg/mL, were selected for subsequent anti-inflammatory assays.

**Figure 6:**
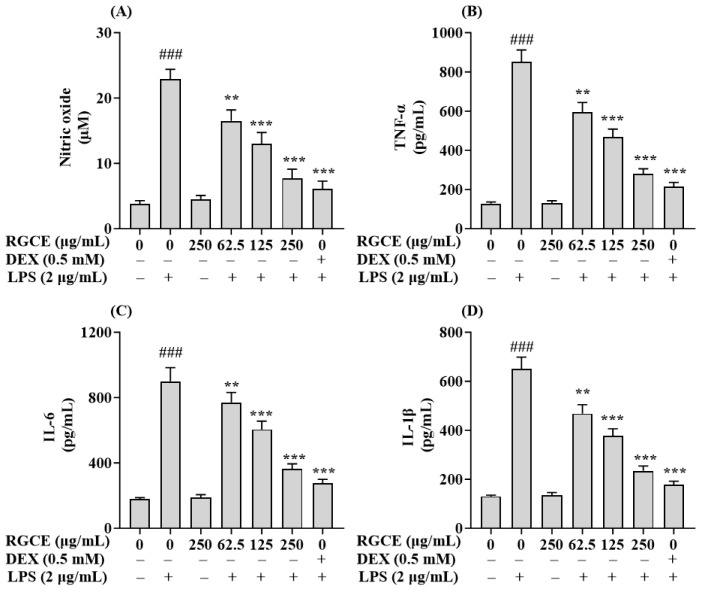
Effects of red ginseng ethanolic extract (RGEE) pretreatment on inflammatory mediator production in LPS-stimulated RAW 264.7 cells. (**A**) Nitric oxide production. (**B**) TNF- level. (**C**) IL-6 level. (**D**) IL-1β level. Data are presented as the mean ± SD (n = 6). ### *p* < 0.001 vs. the Control group; ** *p* < 0.01 and *** *p* < 0.001 vs. the LPS group.

As shown in Figure 6, LPS stimulation markedly increased NO production and the secretion of pro-inflammatory cytokines, including TNF-α, IL-6, and IL-1β, compared with the untreated control group (*p* < 0.001). RGEE pretreatment dose-dependently reduced LPS-induced nitrite production. In addition, RGEE significantly suppressed the LPS-induced increases in TNF-α, IL-6, and IL-1β levels, with the strongest inhibitory effect observed at 250 μg/mL. The DEX-treated group also showed marked reductions in nitrite and pro-inflammatory cytokine levels, confirming the responsiveness of the LPS-induced inflammatory model. These results indicate that RGEE, at non-cytotoxic concentrations, effectively attenuated LPS-induced inflammatory responses in RAW 264.7 macrophages by reducing nitrite production and suppressing pro-inflammatory cytokine secretion.

## 3. Discussion

The DSS-induced colitis model is commonly used to investigate UC-like intestinal inflammation and to evaluate candidate interventions [26]. This model is typically characterized by body weight loss, diarrhea, rectal bleeding, colon shortening, spleen enlargement, and histopathological injury, thereby partially mimicking the clinical and pathological features of human UC [12,27,28]. Previous studies have reported that Korean Red Ginseng (KRG) can ameliorate DSS-induced body weight loss, diarrhea, rectal bleeding, and colon shortening, possibly through the inhibition of inflammation-related signaling pathways, including COX-2, iNOS, NF-κB, and STAT3 [29,30]. Recent studies have also shown that KRG can alleviate DSS-induced colitis by modulating the gut microbiota [24]. These findings indicate that ginseng-derived preparations may exert protective effects against colitis through multiple inflammation-related and intestinal homeostasis-related processes. Based on these findings, the present study further investigated RGEE from an application-oriented perspective. However, whether RGEE also acts through the inflammation-related signaling pathways, epithelial barrier regulation, oxidative stress responses, or gut microbiota modulation reported for other ginseng-derived preparations remains to be further clarified in future studies.

In the present study, DSS treatment induced body weight loss, increased DAI scores, rectal bleeding, colon shortening, and histopathological injury, indicating the successful establishment of experimental colitis. RGEE pretreatment alleviated these abnormalities to varying degrees. H&E staining further showed that RGEE pretreatment improved colonic tissue architecture, reduced crypt damage and inflammatory cell infiltration, and significantly decreased histopathological scores. These findings suggest that RGEE has protective potential against DSS-induced colitis at the tested dose.

Nevertheless, because only one RGEE dose was evaluated in the present study, the current design does not allow determination of the minimum effective dose, optimal dose, therapeutic window, or dose-dependent efficacy. Therefore, the pharmacological interpretation of the present findings should be limited to preliminary evidence obtained at the tested dose. In addition, although histological improvement suggests attenuation of DSS-induced mucosal injury, epithelial barrier function was not directly assessed using tight junction markers such as ZO-1, Occludin, or Claudin-1, mucus-related markers, or intestinal permeability assays. Thus, the observed histological preservation should not be interpreted as direct evidence of epithelial barrier protection. Future studies incorporating multiple RGEE doses, long-term safety evaluation, pharmacokinetic analysis, and mechanism-focused experiments are needed to further clarify the pharmacological characteristics and mechanisms of RGEE.

In addition, previous studies on individual ginsenosides provide useful context for interpreting the potential intestinal protective properties of ginseng-derived preparations. For example, ginsenoside Rc has been reported to improve DSS-induced colitis by regulating inflammatory cytokines, NF-κB signaling, and tight junction-related markers. Other individual or rare ginsenosides, such as ginsenosides Rk3, Rf, and Rb1, have also been shown to alleviate colitis by suppressing inflammatory cytokines [31,32,33,34,35,36]. Unlike these single-compound studies, the present study used a multi-component red ginseng ethanol crude extract. Therefore, the saponin-related LC-MS features detected in RGEE may provide chemical clues for further investigation of its observed biological activity, whereas the direct contribution of individual annotated constituents to the anti-inflammatory effects of RGEE remains to be established.

Hematological parameters and spleen weight provided supplementary information on systemic inflammatory status. DSS treatment increased WBC, NEU, LYM, MON, and PLT levels and enlarged the spleen, suggesting peripheral inflammatory responses and immune organ activation. In addition, DSS decreased erythrocyte-related parameters, including RBC, HGB, and MCH, indicating that DSS-induced colitis may be accompanied by hematological alterations [37,38]. RGEE pretreatment partially improved these DSS-induced hematological changes and significantly decreased spleen weight, indicating that RGEE may alleviate not only local intestinal injury but also DSS-associated systemic inflammatory responses. In addition, RGEE did not cause obvious changes in the weights of other major organs, including the liver, kidneys, lungs, heart, and brain, providing preliminary safety-related observations under the present experimental conditions.

Macrophage-mediated inflammatory responses play an important role in the occurrence and progression of UC. The LPS-stimulated RAW 264.7 macrophage model is commonly used to evaluate the in vitro anti-inflammatory activity of natural products, as LPS stimulation can induce NO production and the production of pro-inflammatory cytokines such as TNF-α, IL-6, and IL-1β [39,40]. In this study, RGEE did not significantly reduce RAW 264.7 cell viability at concentrations ranging from 3.91 to 250 μg/mL. At concentrations of 62.5–250 μg/mL, RGEE dose-dependently reduced LPS-induced NO/nitrite production and significantly suppressed the release of TNF-α, IL-6, and IL-1β. These in vitro findings suggest that RGEE can suppress macrophage-derived inflammatory mediator production, which may partly support its anti-inflammatory potential observed in the DSS-induced colitis model. In the present study, different positive controls were used for the in vivo and in vitro experiments. 5-ASA was used as the positive control in the DSS-induced colitis mouse model, whereas DEX was used only in the LPS-stimulated RAW 264.7 macrophage model. The study was not designed as a formal efficacy comparison between RGEE and these standard anti-inflammatory agents. In vivo, RGEE showed protective effects against DSS-induced colitis, as reflected by improvements in disease activity, colon shortening, histopathological injury, and inflammatory responses, but its efficacy should not be interpreted as equivalent or superior to that of 5-ASA. In vitro, DEX served as a potent anti-inflammatory reference to validate the LPS-induced macrophage inflammatory response, while RGEE exhibited a relatively moderate inhibitory effect on inflammatory mediator production. These findings suggest that RGEE may have potential supportive value as a natural-product-based intervention rather than serving as a direct substitute for standard anti-inflammatory drugs.

LC-MS analysis showed that multiple saponin-related LC-MS features were putatively annotated in RGEE, including notoginsenoside R1, ginsenoside Rb1, ginsenoside Rb2, 20(R)-ginsenoside Rh1, ginsenoside Rh4, 20(R)-ginsenoside Rh2, notoginsenoside Fc, notoginsenoside S, and notoginsenoside Fa. Ginsenosides are important bioactive constituents of ginseng and red ginseng, and their structural diversity and metabolic transformation may influence their anti-inflammatory, immunomodulatory, and intestinal protective activities [5,41,42]. Therefore, the presence of multiple saponin-related LC-MS features in RGEE may provide a preliminary chemical basis for further investigation of its anti-inflammatory and anti-colitis effects. It should be noted that the LC-MS annotations in this study were mainly inferred based on retention time, molecular formula, adduct ions, observed *m*/*z* values, and database matching. Because these annotations were not confirmed using reference standards, potential structural isomers, isobaric compounds, or database-derived false-positive matches cannot be excluded. Further confirmation using reference standards and targeted quantitative analysis is therefore required in future studies.

Compared with single compounds, red ginseng ethanol crude extract (RGEE) is relatively simple to prepare and more closely resembles practical functional food ingredients, natural anti-inflammatory materials, or dietary supplement products, suggesting its potential application value. In the present study, RGEE alleviated DSS-induced colitis phenotypes, improved colonic histopathological injury, modulated systemic inflammation-related parameters, and suppressed macrophage-derived inflammatory mediator production. These findings indicate that RGEE may have potential supportive value as a natural-product-based functional ingredient for UC-related intestinal inflammation.

However, several limitations should be acknowledged. For example, the present study was not designed as a formal efficacy comparison between RGEE and standard anti-inflammatory drugs; therefore, the effects of RGEE should not be interpreted as equivalent or superior to those of 5-ASA or DEX; although RGEE inhibited LPS-induced inflammatory responses in RAW 264.7 macrophages, endotoxin contamination in the extract was not directly evaluated. Because macrophages are highly sensitive to endotoxin exposure, the possibility that trace endotoxin contamination may have influenced inflammatory readouts cannot be excluded; the current study evaluated only one RGEE dose, which limits determination of the minimum effective dose, optimal dose, therapeutic window, and dose-dependent efficacy.

Future studies should therefore prioritize dose–response evaluation, followed by the direct assessment of intestinal barrier function using tight junction markers, mucus-related markers, and intestinal permeability assays. Further validation of NF-κB/MAPK/STAT3 signaling, gut microbiota characterization, pharmacokinetic analysis, long-term safety assessment, and chronic or relapsing colitis models will be needed to clarify the mechanism of action and translational relevance of RGEE.

## 4. Materials and Methods

### 4.1. Plant Materials

Fresh ginseng roots, aged 3–4 years, were purchased from Fusong County, Baishan City, Jilin Province, China. After removing soil and impurities, the samples were washed thoroughly and drained to remove surface moisture. Red ginseng was prepared according to a previously reported method with minor modifications. Briefly, the cleaned ginseng roots were steamed at 95–100 °C for 7 h until they became reddish-brown and semi-transparent. The steamed samples were then dried in an oven at 45 °C to a constant weight. The dried red ginseng was ground into powder, passed through an 80-mesh sieve, packed in airtight polyethylene bags, and stored in a dry and dark environment until further use [16,43].

### 4.2. Chemicals

Dextran sulfate sodium (DSS; molecular weight, [36,000–50,000 Da]; catalog no. 160110; lot no. YD08001) was purchased from MP Biomedicals (Irvine, CA, USA). Lipopolysaccharide (LPS) was purchased from Sigma-Aldrich (St. Louis, MO, USA). Dulbecco’s modified Eagle’s medium (DMEM), fetal bovine serum (FBS), and penicillin-streptomycin solution were obtained from Gibco (Waltham, MA, USA). The Cell Counting Kit-8 (CCK-8) was purchased from Beyotime Biotechnology Co., Ltd. (Shanghai, China). Griess reagent kits and ELISA kits were purchased from Wuhan Elabscience Biotechnology Co., Ltd. (Wuhan, China).

### 4.3. Preparation of Red Ginseng Ethanolic Extract (RGEE)

Red ginseng powder (10 g) was extracted with 40 volumes of 75% ethanol using flash extraction twice, for 60 s each time. The extracts were combined, filtered, and concentrated under reduced pressure. The resulting concentrate was freeze-dried to obtain RGEE, which was stored at −20 °C until further use. In the present study, 4.56 g of freeze-dried RGEE was obtained from 10 g of red ginseng powder, corresponding to an extraction yield of 45.6%.

### 4.4. Component Analysis of Red Ginseng Ethanol Extract

The chemical constituents of RGEE were analyzed using an ultra-performance liquid chromatography quadrupole time-of-flight tandem mass spectrometry system (UPLC-Q-TOF-MS/MS). For sample preparation, 50 μL of sample solution was mixed with 1 mL of pure methanol, vortexed for 10 min, and centrifuged at 12,000 rpm for 10 min. The supernatant was collected for injection.

Chromatographic separation was performed on a Waters BEH C18 column (1.7 μm, 2.1 × 50 mm). The mobile phase consisted of 0.1% formic acid in water as solvent A and 0.1% formic acid in acetonitrile as solvent B. The column temperature was maintained at 40 °C, the flow rate was 0.4 mL/min, and the injection volume was 2 μL. The gradient elution program was as follows: 0–1 min, 95% A and 5% B; 1–35 min, A was linearly decreased from 95% to 2%, while B was linearly increased from 5% to 98%; and 35–40 min, 2% A and 98% B were maintained.

Mass spectrometric detection was performed using an electrospray ionization source (ESI) in MSE continuum mode. Data were acquired in both positive and negative ion modes over an *m*/*z* range of 50–1200. Argon was used as the collision gas and nitrogen as the nebulizing/desolvation gas. The desolvation gas flow rate was 800 L/h in both positive and negative ion modes, and the cone gas flow rate was 50 L/h. The desolvation temperature and ion source temperature were set at 400 °C and 110 °C, respectively. The capillary voltage was 2.0 kV, and the collision energy was set at 20–40 V. Sodium formate was used for mass axis calibration [42,44].

### 4.5. Effects of RGEE on DSS-Induced Colitis in Mice

#### 4.5.1. Experimental Animals

Specific pathogen-free (SPF) male C57BL/6 mice, aged 6–8 weeks and weighing 18–22 g, were purchased from Liaoning Changsheng Biotechnology Co., Ltd. (Benxi, China). The mice were housed in an SPF-grade animal facility at Yanbian University Laboratory Animal Center and acclimatized for 1 week under standard environmental conditions before the subsequent experiments. The housing conditions were as follows: temperature, 20–25 °C; relative humidity, 40–60%; 12 h/12 h light/dark cycle; and free access to a standard pellet diet and sterile drinking water. All animal experimental procedures were performed in strict accordance with the ethical guidelines for laboratory animals and were approved by the Animal Ethics Committee of the corresponding institution.

#### 4.5.2. Experimental Grouping and Sample Collection

A mouse UC model was established using 3% (*w*/*v*) DSS. After 1 week of acclimatization, the mice were randomly divided into four groups, with 10 mice in each group: a normal control group (Control), a DSS-induced disease model group (DSS), an RGEE group (100 mg/kg), and a 5-aminosalicylic acid group (5-ASA, 100 mg/kg; positive control) [28]. The RGEE dose of 100 mg/kg was selected based on previous studies of red ginseng-related preparations, preliminary tolerability observations, and the purpose of conducting an initial proof-of-concept efficacy evaluation. This dose did not cause obvious abnormal behavior, mortality, or apparent changes in major organ weights under the present experimental conditions.

The Control group received an equal volume of vehicle by oral gavage throughout the experiment and had free access to sterile drinking water. The DSS group received an equal volume of sterile water by oral gavage throughout the experiment and was given 3% DSS solution during the modeling period. The RGEE and 5-ASA groups were orally administered RGEE or 5-ASA once daily according to body weight from day 1 of the experiment for 10 consecutive days. From days 4 to 10, the DSS, RGEE, and 5-ASA groups were simultaneously given 3% (*w*/*v*) DSS solution ad libitum to induce colitis.

During the experiment, the general condition of the mice was observed daily at a fixed time, and body weight, food intake, water intake, stool consistency, and rectal bleeding were recorded. After the final administration, the mice were fasted for 12 h with free access to water, and samples were collected under anesthesia. Whole blood was collected for hematological analysis. Major organs were harvested and weighed. The colon was isolated, and its length was measured. A portion of the colon tissue was fixed in 4% paraformaldehyde for hematoxylin and eosin (H&E) staining analysis.

#### 4.5.3. Measurement of Body Weight Change and DAI

During the experiment, the body weight of each mouse was measured daily, and the body weight change rate was calculated using the following formula:

DAI was used to evaluate the severity of DSS-induced colitis in mice. The DAI score consisted of three parameters: body weight change, stool consistency, and rectal bleeding. According to established evaluation criteria for DSS-induced colitis, each parameter was scored separately, and the total DAI score was calculated. Body weight change was scored as 0, 1, 2, 3, or 4. Stool consistency was classified as normal stool, soft stool, loose stool, or diarrhea. Rectal bleeding was scored based on fecal occult blood test results and visible bleeding. A higher DAI score indicated more severe colitis activity.

#### 4.5.4. H&E Staining and Histopathological Scoring

Colon tissues were fixed in 4% paraformaldehyde for 24 h, followed by routine dehydration, clearing, and paraffin embedding. Serial sections with a thickness of 4 μm were prepared. The sections were deparaffinized in xylene, rehydrated through a graded ethanol series, and then sequentially subjected to hematoxylin staining, washing with tap water, differentiation with acid alcohol, bluing, eosin counterstaining, dehydration, clearing, and mounting with neutral resin. Histomorphological changes in colon tissues were observed and photographed under a light microscope. Histopathological scoring was performed using a semi-quantitative scoring system based on mucosal architecture disruption, crypt damage, inflammatory cell infiltration, and goblet cell depletion. Each parameter was scored on a 0–3 scale as follows: 0, no obvious abnormality; 1, mild alteration; 2, moderate alteration; and 3, severe alteration. The total histopathological score was calculated by summing the scores of all parameters, with higher scores indicating more severe colonic injury. H&E-stained sections were independently evaluated by two observers blinded to the experimental groups. Discrepancies between observers were resolved by joint review and consensus.

#### 4.5.5. Hematological Analysis

Hematological parameters were measured using an animal hematology analyzer (Mindray Medical Co., Ltd., Shengzheng, China). The analyzed parameters included white blood cells (WBC), neutrophils (NEU), lymphocytes (LYM), monocytes (MON), red blood cells (RBC), hemoglobin (HGB), hematocrit (HCT), mean corpuscular hemoglobin (MCH), mean corpuscular volume (MCV), mean corpuscular hemoglobin concentration (MCHC), platelets (PLT), and mean platelet volume (MPV). Changes in hematological parameters among the groups were compared to provide supplementary information on peripheral inflammatory responses and hematological status after DSS induction.

### 4.6. Effects of RGEE on LPS-Induced Inflammatory Mediator Production in RAW 264.7 Cells

#### 4.6.1. RAW 264.7 Cell Culture

RAW 264.7 cells were purchased from the American Type Culture Collection (ATCC, Manassas, VA, USA). After resuscitation, RAW 264.7 cells were cultured in complete DMEM supplemented with 10% FBS and 1% penicillin–streptomycin solution. The cells were maintained in a humidified incubator at 37 °C with 5% CO_2_. Cells in good growth condition and at 70–80% confluence were used for subsequent experiments [45].

#### 4.6.2. Cell Viability Assay

RAW 264.7 cells were seeded into 96-well plates at a density of 1.0 × 10^4^ cells per well and cultured overnight. After removing the culture medium, cells in the experimental groups were treated with medium containing different concentrations of RGEE at final concentrations of 3.91, 7.81, 15.62, 31.25, 62.50, 125, 250, 500, and 1000 μg/mL for 24 h. Blank wells containing medium without cells were used for background correction, while control cells were treated with fresh medium without RGEE.

After treatment, 10 μL of CCK-8 solution was added to each well, followed by incubation for 1 h. The absorbance was measured at 450 nm (Multiskan FC, Thermo Fisher Scientific Co., Waltham, MA, USA). Cell viability was calculated based on the absorbance values to evaluate the effect of RGEE on RAW 264.7 cell viability and to determine safe concentrations for subsequent anti-inflammatory experiments.

#### 4.6.3. Determination of NO Production

RAW 264.7 cells were seeded into 48-well plates at a density of 2.0 × 10^5^ cells/well in 300 μL of culture medium and cultured overnight for 12 h. After removing the medium, the Control and LPS groups were treated with DMEM without RGEE. The RGEE-treated groups were treated with RGEE at concentrations of 62.5, 125, and 250 μg/mL, and the positive control group was treated with 0.5 mM dexamethasone (DEX). After pretreatment for 1 h, LPS was added to all groups except the Control group at a final concentration of 2 μg/mL, followed by incubation at 37 °C for 24 h.

After incubation, the culture supernatants were collected, and NO levels were measured using a Griess reagent kit according to the manufacturer’s instructions. The absorbance was measured at 550 nm, and NO concentrations were calculated based on a standard curve.

#### 4.6.4. Determination of Inflammatory Cytokines

RAW 264.7 cells were seeded into 48-well plates at a density of 2.0 × 10^5^ cells/well in 300 μL of culture medium and cultured overnight for 12 h. After removing the medium, the Control and LPS groups were treated with culture medium without RGEE. The RGEE-treated groups were treated with RGEE at concentrations of 62.5, 125, and 250 μg/mL, and the positive control group was treated with 0.5 mM DEX. After pretreatment for 1 h, LPS was added to all groups except the Control group at a final concentration of 2 μg/mL, followed by incubation for 24 h.

After incubation, the culture supernatants were collected into 1.5 mL centrifuge tubes and stored at −20 °C until analysis. The secretion levels of TNF-α, IL-1β, and IL-6 in the culture supernatants were measured using ELISA kits.

### 4.7. Statistical Analysis

All experimental data are presented as the mean ± SD. Statistical analyses were performed using GraphPad Prism 10.1 or SPSS 23.0 software. Data normality and homogeneity of variance were assessed using the Shapiro–Wilk test and Levene’s test, respectively. For endpoint measurements, normally distributed data with homogeneous variance were analyzed by one-way ANOVA followed by pre-planned multiple comparisons with Bonferroni correction; otherwise, appropriate non-parametric tests were used. For time-course variables, including body weight and disease activity index (DAI), repeated-measures ANOVA or a mixed-effects model was applied to evaluate treatment, time, and treatment × time interaction effects. In animal experiments, the DSS group was compared with the Control group to confirm model induction, whereas the RGEE and 5-ASA groups were compared with the DSS group to evaluate treatment effects. In cell experiments, the LPS group was compared with the Control group to confirm inflammatory model induction, whereas the RGEE- and DEX-treated groups were compared with the LPS group to evaluate anti-inflammatory effects. Statistical significance was set at *p* < 0.05.

## 5. Conclusions

This study demonstrated that RGEE alleviated DSS-induced colitis symptoms in mice, as evidenced by reduced body weight loss, decreased disease activity index, alleviated rectal bleeding, partially restored colon length, and attenuated colonic histopathological injury. Hematological parameters and spleen weight further suggested that RGEE may modulate DSS-induced peripheral inflammatory responses and systemic immune activation. In vitro, RGEE suppressed LPS-induced NO/nitrite, TNF-α, IL-6, and IL-1β production in RAW 264.7 macrophages at non-cytotoxic concentrations, indicating that its anti-colitis effect may be associated with the inhibition of inflammatory mediator release. LC-MS analysis revealed multiple saponin-related constituents in RGEE, which may provide a chemical basis for its anti-inflammatory and intestinal protective activities. Overall, RGEE may serve as a potential functional ingredient for the prevention or adjunctive management of UC, although further studies are required to confirm its major active constituents, molecular mechanisms, and long-term safety.

## Figures and Tables

**Table 1:** Saponin constituents tentatively identified in RGEE by LC-MS analysis.

No.	RT (min)	Molecular Formula	Adduction	Observed *m*/*z*	Relative Response (%)	Major MS/MS Fragmentations (*m*/*z*)	Tentatively Identified Compound
1	7.79	C_36_H_62_O_10_	[M + HCOO]^−^	699.43	0.25	653.43, 491.37	(24S)-Pseudoginsenoside RT4
2	11.27	C_63_H_106_O_30_	[M + HCOO]^−^	1387.67	1.09	1341.67, 1209.63, 1077.59	Notoginsenoside S
3	11.50	C_41_H_66_O_11_	[M + H]^+^	735.47	0.20	735.47, 589.41, 455.35	Eleutheroside K
4	11.94	C_36_H_60_O_8_	[M + H]^+^	621.44	1.94	621.44, 459.38, 441.37	Ginsenoside Rh4
5	11.96	C_54_H_92_O_23_	[M + HCOO]^−^	1153.60	24.87	1107.60, 945.54, 783.49	Ginsenoside Rb1
6	12.20	C_36_H_62_O_9_	[M + HCOO]^−^	683.44	10.52	637.43, 475.38	20(R)-Ginsenoside Rh1
7	12.21	C_53_H_90_O_22_	[M + HCOO]^−^	1123.59	13.54	1077.58, 945.54, 915.53	Ginsenoside Rb2
8	12.29	C_58_H_98_O_26_	[M + HCOO]^−^	1255.63	7.93	1209.63, 1077.59, 945.54	Notoginsenoside Fc
9	13.27	C_59_H_100_O_27_	[M + HCOO]^−^	1285.65	0.12	1239.64, 1107.59, 1077.58	Notoginsenoside Fa
10	13.34	C_47_H_80_O_18_	[M + CH_3_COO]^−^	991.55	39.10	931.53, 799.48, 637.43	Notoginsenoside R1
11	17.01	C_36_H_62_O_8_	[M + HCOO]^−^	667.44	0.21	621.44, 459.38, 441.37	20(R)-Ginsenoside Rh2
12	18.32	C_36_H_58_O_8_	[M + HCOO]^−^	663.41	0.21	617.40, 455.35	Oleanolic acid 28-O-β-D- glucopyranoside

Compounds were tentatively identified based on LC-MS analysis and database matching.

**Table 2:** Effects of red ginseng ethanolic extract (RGEE) pretreatment on hematological parameters in mice with DSS-induced colitis.

Group	WBC (10^9^/L)	NEU (10^9^/L)	LYM (10^9^/L)	MON (10^9^/L)
Control	7.22 ± 2.26	1.60 ± 0.82	4.20 ± 2.18	0.19 ± 0.05
DSS	13.57 ± 1.10 ^###^	4.15 ± 0.73 ^###^	8.90 ± 0.74 ^###^	0.47 ± 0.15 ^###^
RGEE	7.40 ± 3.13 ***	1.79 ± 0.62 ***	4.61 ± 2.23 ***	0.26 ± 0.13 ***
5-ASA	7.29 ± 2.47 ***	1.66 ± 0.93 ***	4.22 ± 1.21 ***	0.20 ± 0.04 ***
	RBC (10^12^/L)	HGB (g/L)	HCT (%)	MCHC (Pg)
Control	10.83 ± 0.42	178.20 ± 7.84	50.7 ± 2.16	16.42 ± 0.10
DSS	8.32 ± 2.94 ^#^	161.58 ± 7.63 ^###^	48.00 ± 14.25 ^ns^	12.80 ± 5.00 ^#^
RGEE	10.55 ± 1.57 *	169.40 ± 7.56 ^ns^	47.38 ± 7.38 ^ns^	16.24 ± 0.17 *
5-ASA	10.78 ± 0.96 *	176.20 ± 3.75 ***	49.20 ± 3.36 ^ns^	16.98 ± 0.29 **
	MCV (fL)	MCHC (g/L)	MPV (fL)	PLT (10^9^/L)
Control	46.82 ± 0.50	350.80 ± 3.84	6.42 ± 0.46	845.18 ± 33.86
DSS	48.01 ± 1.66 ^ns^	350.69 ± 4.90 ^ns^	6.39 ± 2.10 ^ns^	1049.60 ± 45.52 ^###^
RGEE	49.52 ± 1.14 ^ns^	352.80 ± 7.44 ^ns^	6.36 ± 0.43 ^ns^	871.20 ± 17.76 ***
5-ASA	46.82 ± 1.35 ^ns^	351.60 ± 3.44 ^ns^	6.38 ± 0.14 ^ns^	838.60 ± 16.88 ***

DSS, DSS-induced colitis model group; 5-ASA, positive control group. WBC = white blood cell count; NEU = neutrophil count; LYM = lymphocyte count; MON = monocyte count; RBC = red blood cell count; HGB = hemoglobin; HCT = hematocrit; MCV = mean corpuscular volume; MCHC = mean corpuscular hemoglobin concentration; MPV = mean platelet volume; PLT = platelet count. Data are presented as the mean ± SD (n = 10). ns, not significant. # *p* < 0.05 and ### *p* < 0.001 vs. the Control group; * *p* < 0.05, ** *p* < 0.01 and *** *p* < 0.001 vs. the DSS group.

## Data Availability

The original contributions presented in this study are included in the article/Supplementary Material. Further inquiries can be directed to the corresponding author.

## Supplementary Materials

The following supporting information can be downloaded at https://www.mdpi.com/article/10.3390/ijms27125325/s1.
