# Supplementary material for: Red Ginseng Ethanolic Extract Alleviates DSS-Induced Colitis in Mice by Suppressing Inflammatory Mediator Production
Source: Int J Mol Sci. 2026 Jun 12;27(12):5325. doi: 10.3390/ijms27125325 (PMC13299471; doi:10.3390/ijms27125325)
Supplement: Supplementary file 1 [file ijms-27-05325-s001.zip › ijms-4338776-supplementary.pdf]

(A)

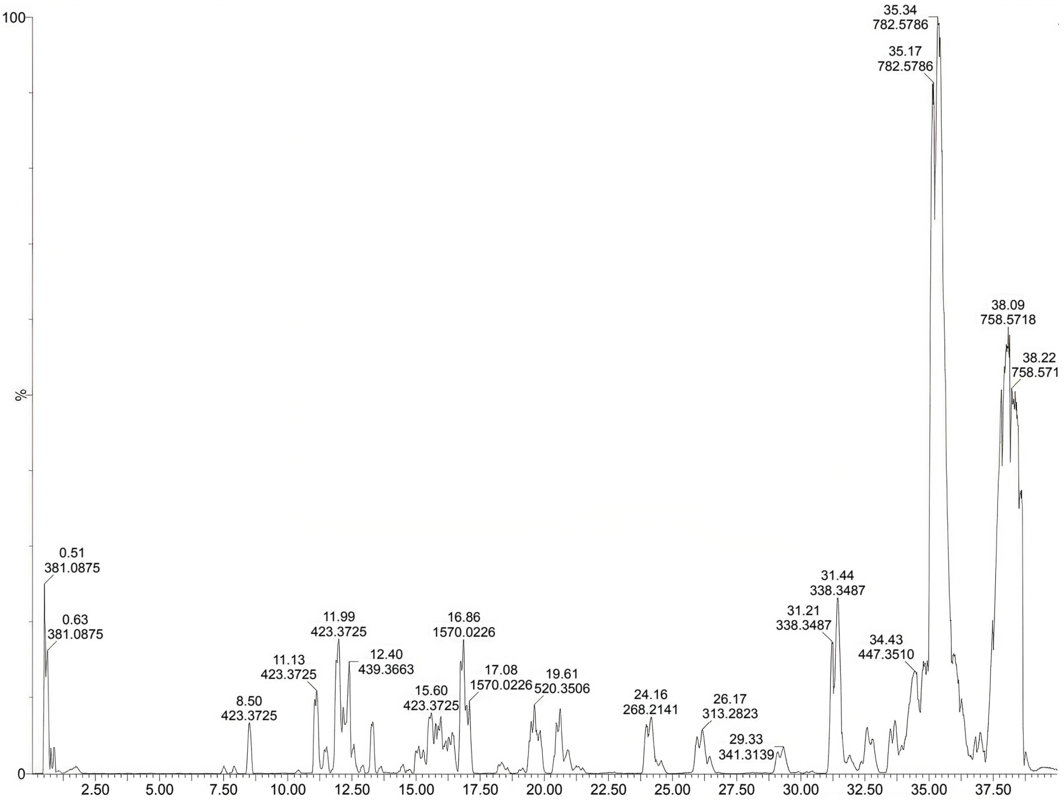

(B)

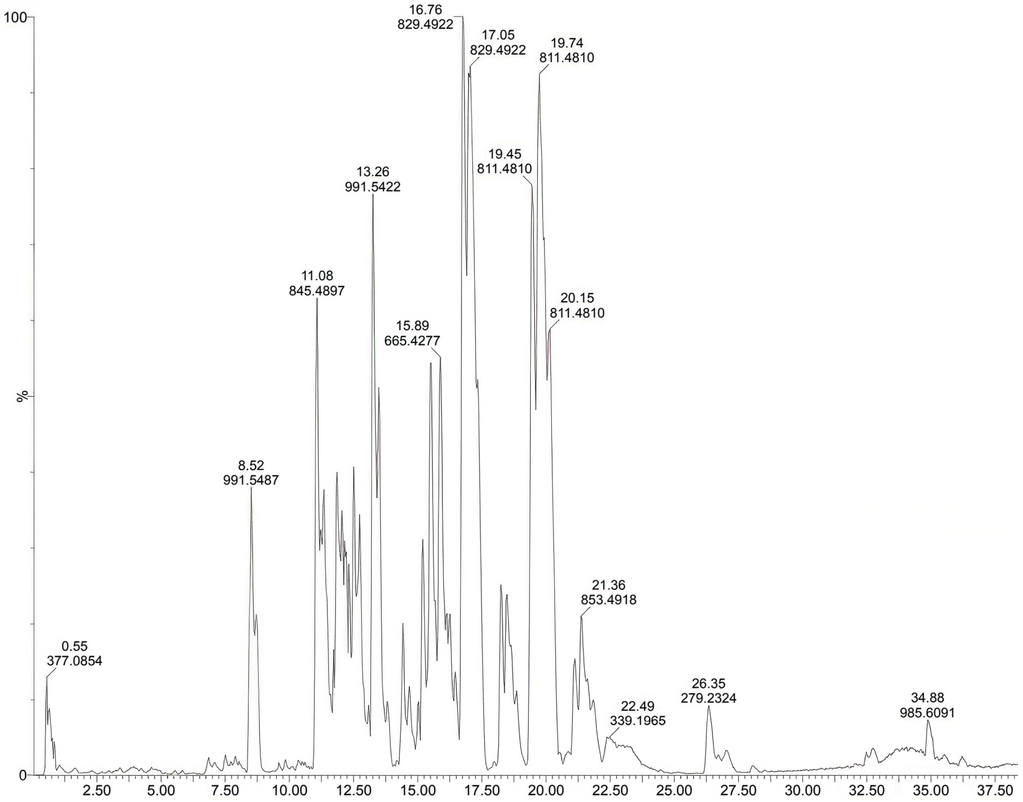

Figure S1 The base peak ion chromatograms of red ginseng ethanolic extract (RGEE) compounds by LC/MS analysis in positive (A) and negative (B) ion modes.
